# miR-92a-3p promotes ox-LDL induced-apoptosis in HUVECs via targeting SIRT6 and activating MAPK signaling pathway

**DOI:** 10.1590/1414-431X20209386

**Published:** 2021-01-15

**Authors:** Yingchun Xu, Chunbo Miao, Jinzhen Cui, Xiaoli Bian

**Affiliations:** 1 The Second People's Hospital of Liaocheng, Department of Cardiology, LiaochengShandong China Department of Cardiology, The Second People's Hospital of Liaocheng, Liaocheng, Shandong, China; 2 The Second People's Hospital of Liaocheng, Department of Internal Medicine, LiaochengShandong China Department of Internal Medicine, The Second People's Hospital of Liaocheng, Liaocheng, Shandong, China; 3 Yangzhou Jiangdu People's Hospital, Jiangdu District, Department of Cardiology, YangzhouJiangsu China Department of Cardiology, Yangzhou Jiangdu People's Hospital, Jiangdu District, Yangzhou, Jiangsu, China

**Keywords:** SIRT6, miR-92a-3p, ox-LDL, HUVECs, Apoptosis

## Abstract

Atherosclerosis could be induced by multiple factors, including hypertension, hyperlipidemia, and smoking, and its pathogenesis has not been fully elucidated. MicroRNAs have been shown to possess great anti-atherosclerotic potential, but the precise function of miR-92a-3p in atherosclerosis and its potential molecular mechanism have not been well clarified. Flow cytometry assay and 3-(4,5-dimethylthiazol-2-yl)-2,5-diphenyl-2H-tetrazol-3-ium bromide (MTT) assay were performed to evaluate effects of oxidized low-density lipoprotein (ox-LDL) on proliferation and apoptosis of human umbilical vein endothelial cells (HUVECs), respectively. Malondialdehyde and superoxide dismutase levels in cell lysate were assessed with biochemical kits. The expression levels of miR-92a-3p and Sirtuin6 (SIRT6) in HUVECs exposed to ox-LDL were estimated by real-time quantitative polymerase chain reaction (RT-qPCR). In addition, the protein levels of SIRT6, c-Jun N-terminal kinase (JNK), phosphorylation JNK (p-JNK), p38 mitogen activated protein kinase (p38 MAPK), and phosphorylation p38 MAPK (p-p38 MAPK) were measured by western blot assays. The relationship between miR-92a-3p and SIRT6 was confirmed by dual-luciferase reporter assay. Ox-LDL induced apoptosis and oxidative stress in HUVECs in concentration- and time-dependent manners. Conversely, miR-92a-3p silencing inhibited apoptosis and SIRT6 expression in HUVECs. The overexpression of miR-92a-3p enhanced apoptosis and phosphorylation levels of JNK and p38 MAPK as well as inhibited proliferation in ox-LDL-induced HUVECs. In addition, SIRT6 was a target of miR-92a-3p. miR-92a-3p negatively regulated SIRT6 expression in ox-LDL-induced HUVECs to activate MAPK signaling pathway *in vitro*. In summary, miR-92a-3p promoted HUVECs apoptosis and suppressed proliferation in ox-LDL-induced HUVECs by targeting SIRT6 expression and activating MAPK signaling pathway.

## Introduction

Atherosclerosis is responsible for stroke and coronary artery diseases worldwide, and is involved in inflammation, lipid metabolism, and oxidative stress (1,2). The vascular endothelium has important functional effects on stimulating response and maintaining vascular homeostasis. Endothelial injury and dysfunction are considered the base and the initial steps for atherosclerosis (3). In inflammatory or other conditions, cell infiltration, endothelial cells apoptosis, and neointima formation are major contributors to the development of atherosclerosis (4,5).

Several studies have recognized the essential role of non-coding RNAs in coronary artery disease, including microRNAs (miRNAs) (6). Not surprisingly, studies have shown that miRNAs could act as suitable tools for diagnosis and therapy of cardiovascular diseases (7). Published reports revealed that miRNAs are associated with the metabolism of lipoproteins that are very stable in body fluids (8,9). Studies have reported that blood miRNAs could function as biomarkers for coronary artery disease (10). For instance, Gao et al. (11) found that miR-122 level is increased in plasma from coronary artery disease patients. Niculescu et al. (12) also identified that circulating miR-486 had a close relationship with some lipid metabolism biomarkers. In addition, a previous report showed that high-density lipoprotein (HDL)-transferred miRNA-223 regulated intercellular adhesion molecule-1 expression to reduce inflammation in primary human coronary aortic endothelial cells (13). Because of limited data, the precise relationship between miRNA and lipid metabolism on coronary artery disease is largely unknown (14).

Oxidized low-density lipoprotein (ox-LDL), a fundamental and major factor for atherosclerosis, largely contributes to apoptosis and increases expression of adhesion molecules in vascular endothelium cells (4). Nàgre-Salvayre et al. (15) reported that ox-LDL was a main contributing factor for injury and apoptosis of endothelial cells by destroying the redox balance of vascular endothelial cells. Another study also found similar results that ox-LDL enhanced autophagy and apoptosis of vascular endothelial cells *in vivo* (16). Ox-LDL has been identified to contribute to the progression of atherosclerosis via inducing apoptosis of vascular endothelial cells (17,18).

Ox-LDL-induced human umbilical vein endothelial cells (HUVECs) has been used as a model for atherosclerosis in many previous studies (19,20). We also established an atherosclerosis model *in vitro* by ox-LDL. Collectively, this research was designed to probe the function of miR-92a-3p in ox-LDL-induced apoptosis of HUVECs.

## Material and Methods

### Cell culture

HUVECs were obtained from American Type Culture Collection (USA) and cultured in RPMI-1640 medium (Gibco BRL, USA) supplemented with 10% (v/v) fetal bovine serum (Gibco BRL) in an atmosphere at 37°C with 5% CO_2_. Ox-LDL was purchased from Sigma (USA) for cell culture.

### Construction of plasmids and transfection

miR-92a-3p mimic, mimic-negative control (NC), miR-92a-3p inhibitor, inhibitor-NC, small interfering RNA (siRNA) against Sirtuin6 (SIRT6) (si-SIRT6), si-NC, overexpression plasmid of SIRT6 (pcDNA3.1-SIRT6), and pcDNA3.1 were purchased from RiboBio (China). The above oligonucleotides or plasmid vectors were transfected into HUVECs by Lipofectamine 2000 reagent (Thermo Fisher Scientific, USA) according to the manufacturer's protocol.

### Flow cytometry

Transfected HUVECs were seeded into a 6-well plate (3.5×10^4^ cells/well) and incubated with various concentrations ox-LDL for 48 h or 50 µg/mL of ox-LDL (given that 100 µg/mL ox-LDL led to approximately 30% apoptosis rate, which may be detrimental to the collection of cells for subsequent gene expression analysis, while 50 µg/mL of ox-LDL caused nearly 50% growth inhibition of HUVECs) for specific times to investigate the effect of ox-LDL on HUVECs. Subsequently, HUVECs were harvested and stained with 5 μL of propodeum iodide (Thermo Fisher Scientific) and 5 μL of annexin V-fluorescein isothiocyanate (Thermo Fisher Scientific) at room temperature for 15 min. The number of apoptotic cells was detected under a flow cytometer (Applied Biosystems, USA).

### 3-(4,5-dimethylthiazol-2-yl)-2,5-diphenyl-2H-tetrazol-3-ium bromide (MTT) assay

In brief, after being seeded into the 96-well plates overnight, HUVECs were incubated with ox-LDL. Next, a total of 20 μL of 5 mg/mL MTT (Thermo Fisher Scientific) was added to each well and incubated for another 4 h. Then, dimethyl sulfoxide (DMSO) was utilized to dissolve the formazan crystals in the 96-well plates. Cell viability was examined by detecting absorbance at 490 nm wavelength on a microplate reader (Applied Biosystems).

### Determination of malondialdehyde (MDA) concentration and superoxide dismutase (SOD) activity

MDA activity was measured by specific assay kits from Beyotime Institute of Biotechnology (China). In brief, HUVECs were incubated with ox-LDL for 48 h. Then, HUVECs were harvested and lysed by phosphate buffered saline. The 100-µL supernatant was collected to react with working fluid and incubated at 37°C for 20 min according to the manufacturer’s instructions. Finally, absorbance was measured at 532 nm to examine the MDA content. In addition, SOD assay was conducted by SOD assay kit (Weishi-Bohui Chromtotech Co, China) according to the manufacturer's protocol. The supernatant (20 µL) was mixed with 180 µL of NBT working buffer and incubated at 37°C for 20 min. Absorbance at 560 nm was detected to assess the SOD activity.

### RNA isolation and real-time quantitative polymerase chain reaction (RT-qPCR)

Total RNA was extracted from HUVECs by Trizol reagent (Qiagen, Germany). Purity of the RNA was assessed by measuring the ratio of the absorbance at 260 and 280 nm. The complementary DNA was synthesized by Prime Script RT Reagent kit (Takara, China) or microRNA Reverse Transcription kit (Thermo Fisher Scientific) in RT-PCR instrument (Applied Biosystems). RT-qPCR assay was performed with Platinum SYBR Green qPCR SuperMix-UDG kit (Invitrogen, USA). Relative expression of genes was calculated and quantified by 2^−ΔΔCt^ method. Glyceraldehyde-3-phosphate dehydrogenase (GAPDH) or endogenous small nuclear RNA U6 was used as internal control. The sequences of primers were: miR-92a-3p (forward, 5′- CTCAACTGGTGTCGTGGAGTCGGCAATTCAGTTGATACAGGCCG-3′; reverse, 5′-ACACTCCAGCTGGGTATTGCACTTGTCCC-3′); SIRT6 (forward, 5′-AAGCTGGAGCCCAAGGAGGAA-3′; reverse, 5′-AAGAATGTGCCAAGTGTAAGA-3′); GAPDH (forward, 5′-TCCCATCACCATCTTCCAGG-3′; reverse, 5′-GATGACCCTTTTGGCTCCC-3′); U6 (forward, 5′-AACGCTTCACGAATTTGCGT-3′; reverse, 5′-CTCGCTTCGGCAGCACA-3′).

### Western blot assay

The transfected HUVECs were lysed with cell lysis buffer (Thermo Fisher Scientific) for total protein extraction. The concentration of protein was measured by a Bradford Protein Assay kit (Sangon Biotech, China). After vortexing for 5 min, equal amounts of protein from each sample were subjected to sodium dodecyl sulfate polyacrylamide gel electrophoresis (SDS-PAGE) and then transferred onto polyvinylidene fluoride (PVDF) membranes (Thermo Fisher Scientific). The membranes were blocked with 5% non-fat milk and probed with antibodies against SIRT6, JNK, p-JNK, p38 MAPK, or p-p38 MAPK (1:1000, Santa Cruz Bio-Technology, USA) overnight at 4°C. Then, the membranes were incubated with the secondary antibody (1:5000, Santa Cruz Bio-technology) with horseradish peroxidase conjugated antibody for 2 h at room temperature. Finally, signal intensity of complexes was visualized with an enhanced chemiluminescence detection kit (Beyotime).

### Dual-luciferase reporter assay

The wild type (wt) 3′UTR segments of SIRT6 containing binding site for miR-92a-3p and its mutant (mut) were inserted into pGL3 (RiboBio), named as SIRT6 wt or SIRT6 mut, respectively. Then HUVECs were transfected with reporter vectors and miR-92a-3p mimics or mimics-NC by Lipofectamine 2000 (Thermo Fisher Scientific) in accordance with instructions. HUVECs were harvested at 24 h post-transfection for the luciferase reporter assay (ratio of firefly and renilla) by Dual-Luciferase Reporter Assay System (Thermo Fisher Scientific).

### Statistical analysis

All statistical analyses were done by SPSS 19.0 statistical software (IBM, USA) with Student's *t*-test or one-way analysis of variance followed by *post hoc* test LSD (Least Significant Difference). A P value less than 0.05 was considered to be statistically significant. Quantitative data are reported as means±SD.

## Results

### Ox-LDL induced apoptosis in HUVECs

**Figure 1:**
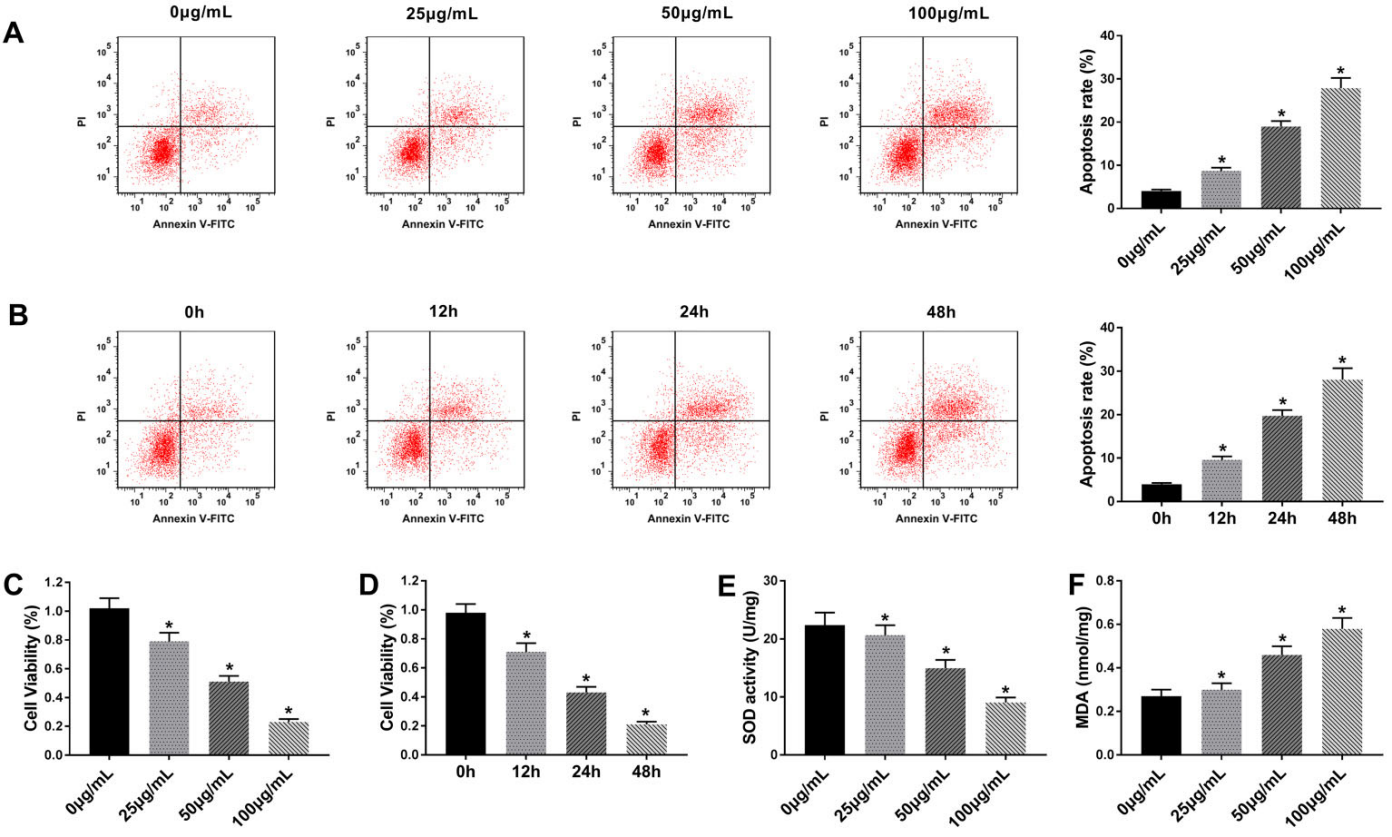
Oxidized low-density lipoprotein (ox-LDL) induced apoptosis and oxidative stress in human umbilical vein endothelial cells (HUVECs). **A** and **B**, Apoptosis rate was assessed by flow cytometry assay after treatment with ox-LDL (0, 25, 50, and 100 µg/mL for 48 h or 50 µg/mL of ox-LDL for 0, 12, 24, and 48 h). **C** and **D**, MTT assay was performed to measure the cell viability of HUVECs treated with ox-LDL. **E** and **F**, Superoxide dismutase (SOD) activity and malondialdehyde (MDA) concentration in cell lysates were analyzed. Data are reported as means±SD with three replicates. *P<0.05 compared to control (ANOVA).

As shown in Figure 1A and B, incubation of HUVECs with 100 µg/mL ox-LDL resulted in the highest apoptosis rate in all treatment groups; furthermore, ox-LDL induced apoptosis of HUVECs in a time-dependent manner. Subsequently, MTT assay suggested that ox-LDL inhibited proliferation of HUVECs in a dose/time-dependent manner (Figure 1C and D). Additionally, ox-LDL inhibited SOD activity and decreased MDA level in HUVECs (Figure 1E and F). Therefore, the results indicated that ox-LDL induced cell apoptosis of HUVECs.

### SIRT6/MAPK signaling pathway was activated in ox-LDL-treated HUVECs

**Figure 2:**
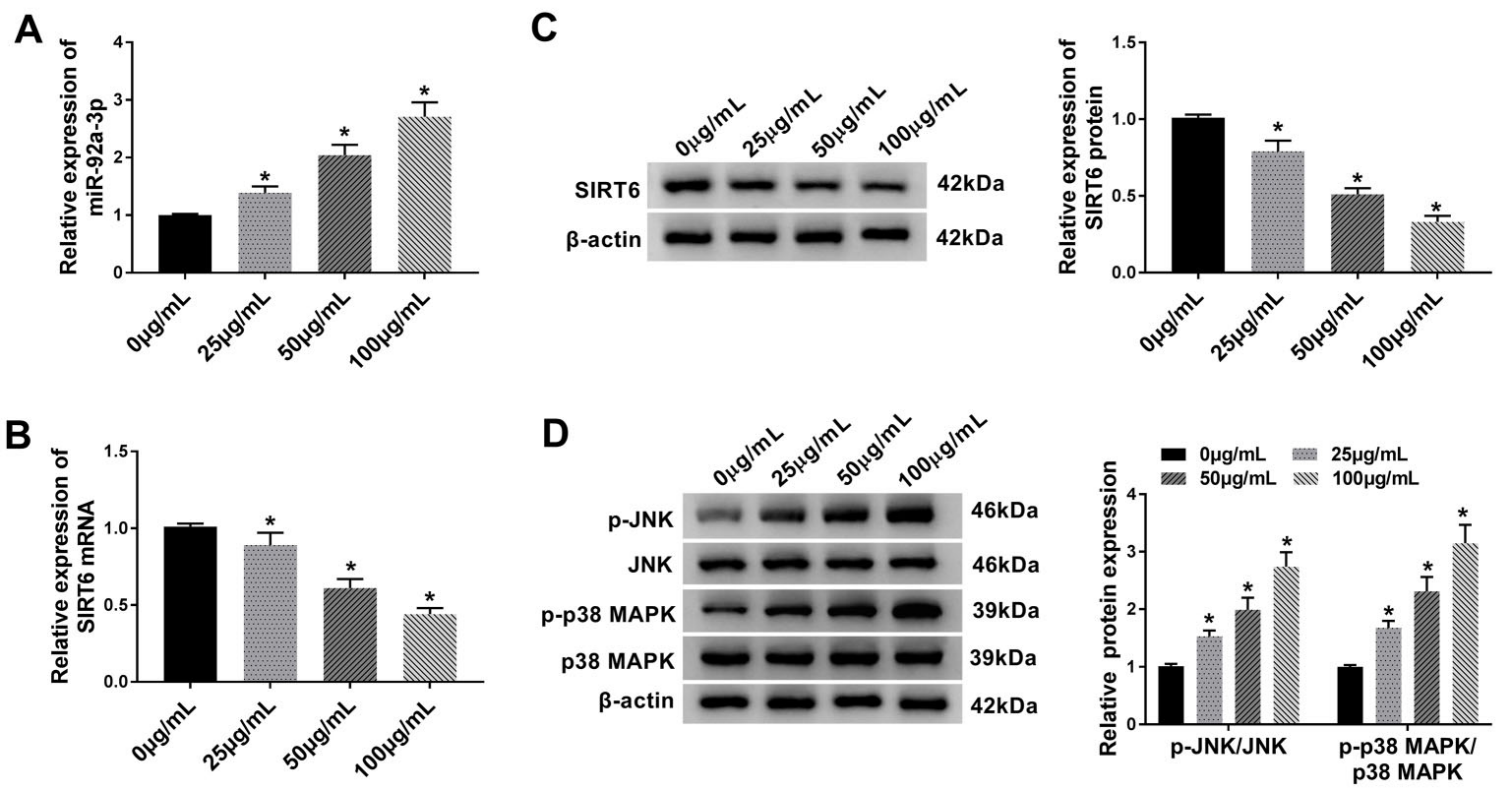
Expression levels of miR-92a-3p and SIRT6 in oxidized low-density lipoprotein (ox-LDL)-treated human umbilical vein endothelial cells (HUVECs). **A**, The relative expression level of miR-92a-3p was evaluated by RT-qPCR assay in ox-LDL-treated HUVECs. **B** and **C**, RT-qPCR and western blot assays were utilized to determine the mRNA and protein expression levels of SIRT6. **D**, Protein expression levels of p-JNK/JNK and p-p38 MAPK/p38 MAPK were analyzed with western blot assays in HUVECs. Data are reported as means±SD with three replicates. *P<0.05 compared to control (ANOVA).

Previous studies revealed the important function of miR-92a and SIRT6 in ox-LDL-treated HUVECs (21,22). We also found that the miR-92a-3p level was increased in ox-LDL-treated HUVECs (Figure 2A). Furthermore, ox-LDL significantly decreased SIRT6 expression in HUVECs in a concentration-dependent manner (Figure 2B and C). Moreover, treatment with ox-LDL increased the ratio of p-JNK/JNK and p-p38 MAPK/p38 MAPK (Figure 2D). Collectively, the results indicated that SIRT6 played a key role in ox-LDL-induced apoptosis of HUVECs. HUVECs were treated with 50 µg/mL of ox-LDL for 48 in the next experiments.

### MiR-92a-3p mediated ox-LDL-induced cell apoptosis by affecting MAPK signaling pathway

**Figure 3:**
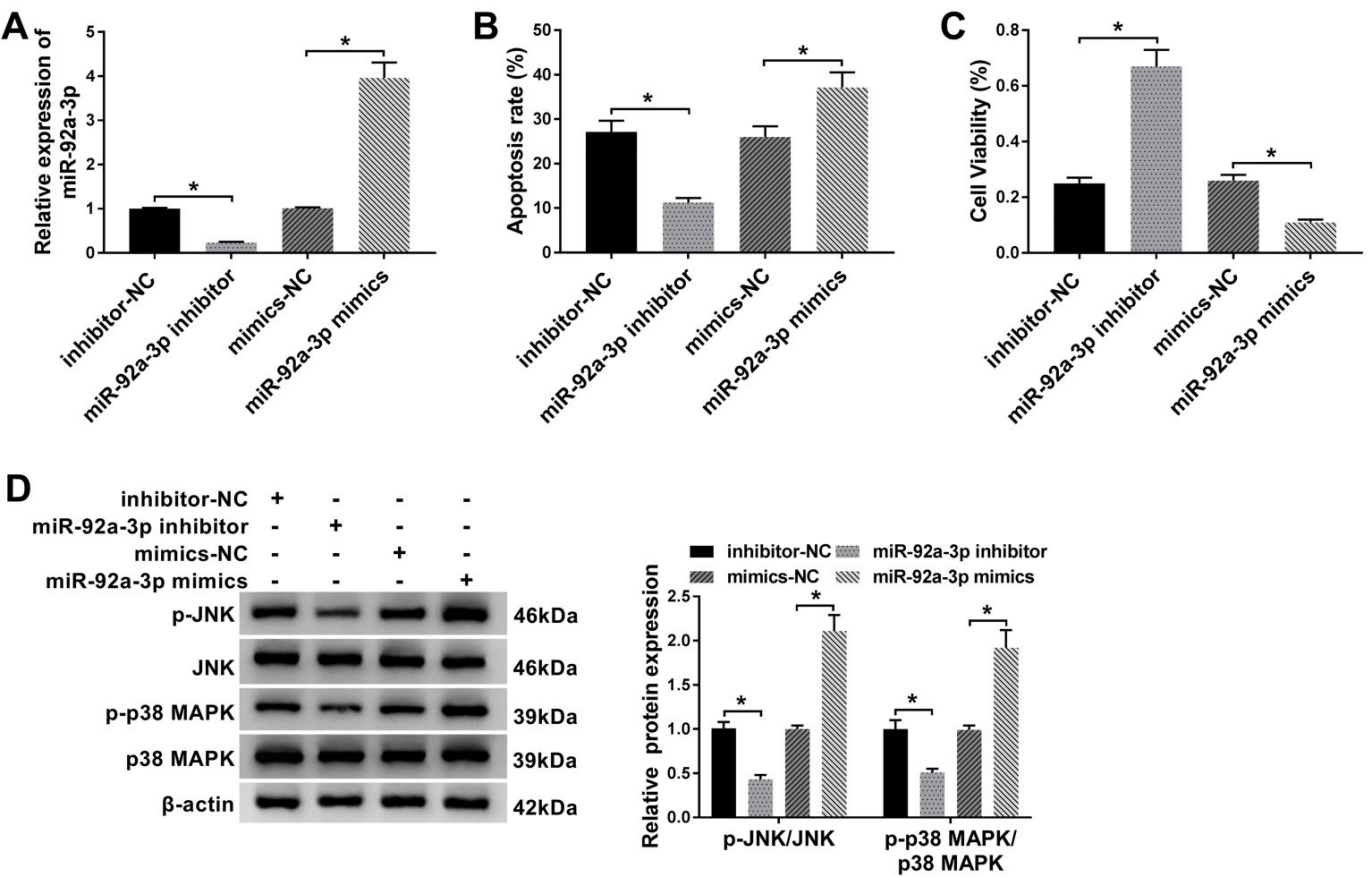
Functional roles of miR-92a-3p in oxidized low-density lipoprotein (ox-LDL)-treated human umbilical vein endothelial cells (HUVECs). HUVECs were treated with 50 µg/mL of ox-LDL for 48 h and transfected with inhibitor-negative control (NC), miR-92a-3p inhibitor, mimics-NC, or miR-92a-3p mimics. **A**, RT-qPCR assay was carried out to evaluate the expression level of miR-92a-3p in HUVECs. **B**, Apoptosis of HUVECs was determined by flow cytometry assay. **C**, MTT analysis was utilized to assess cell viability in HUVECs. **D**, The expression levels of p-JNK/JNK and p-p38 MAPK/p38 MAPK were measured by western blot assays. Data are reported as means±SD with three replicates. *P<0.05 (ANOVA).

Transfection efficiency was confirmed by RT-qPCR assay in HUVECs. Transfection with miR-92a-3p inhibitor inhibited miR-92a-3p expression, while transfection with miR-92a-3p mimics increased miR-92a-3p expression in HUVECs (Figure 3A). The data of flow cytometry assay showed that silencing of miR-92a-3p inhibited apoptosis while overexpression of miR-92a-3p induced apoptosis in HUVECs exposed to ox-LDL (Figure 3B). In addition, miR-92a-3p silencing enhanced cell viability compared with the inhibitor-NC group, whereas miR-92a-3p mimics led to an opposite result (Figure 3C). Western blot assay revealed that the ratio of p-JNK/JNK and p-p38 MAPK/p38 MAPK were significantly increased in the miR-92a-3p mimics group compared to the control group, whereas miR-92a-3p inhibition decreased the ratio of p-JNK/JNK and p-p38 MAPK/p38 MAPK in HUVECs (Figure 3D). Together, these results suggested a close relationship between miR-92a-3p and MAPK signaling pathway in ox-LDL-induced HUVECs.

### MiR-92a-3p negatively targeted SIRT6

**Figure 4:**
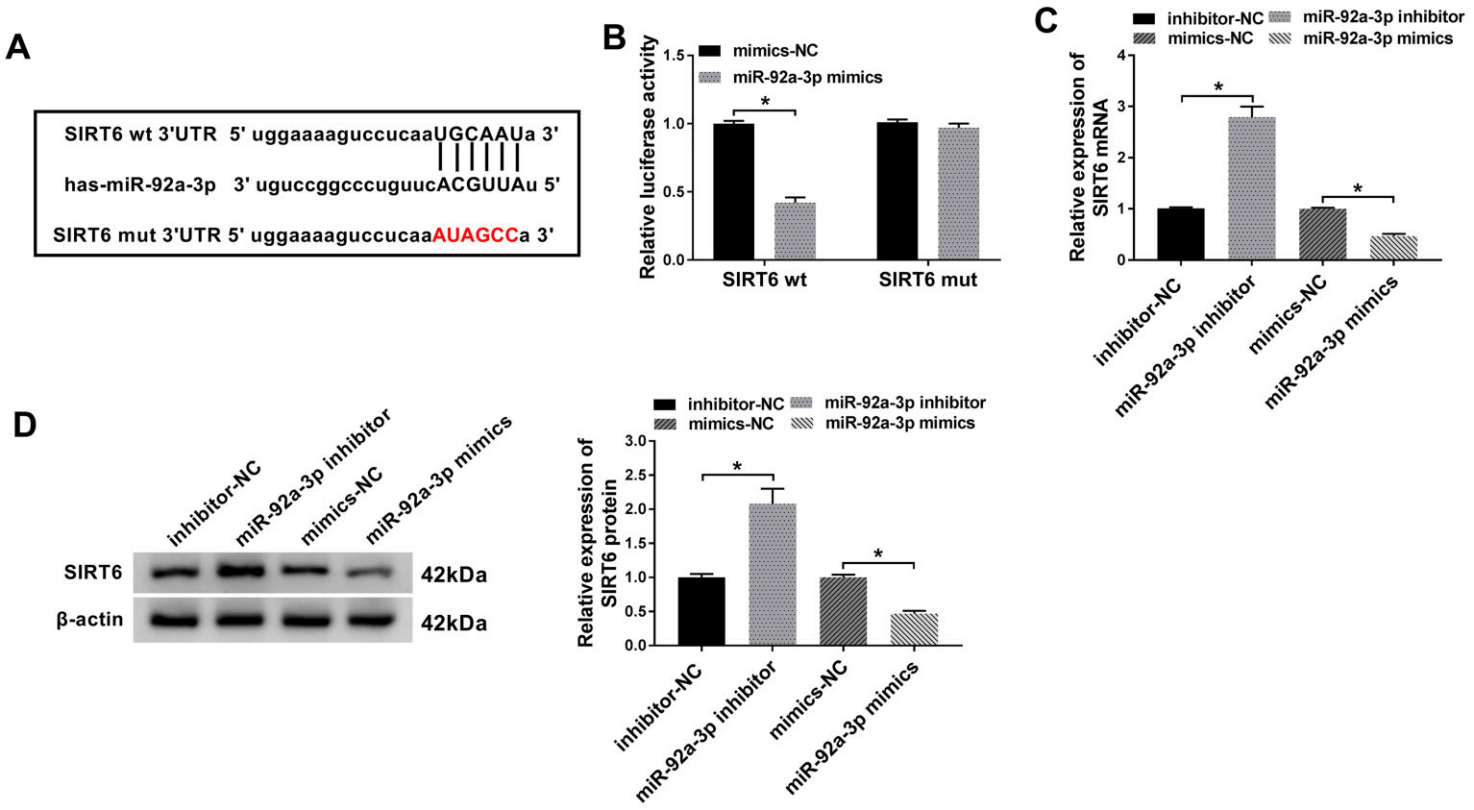
SIRT6 was a target of miR-92a-3p. **A**, Putative binding sites of miR-92a-3p on 3'UTR of SIRT6. **B**, Luciferase activities were measured in human umbilical vein endothelial cells (HUVECs) co-transfected with SIRT6 wild type and mutant (wt/mut) luciferase reporter and miR-92a-3p mimics/miR-negative control (NC). **C** and **D**, Protein and mRNA levels of SIRT6 were assessed by RT-qPCR and western blot assays in HUVECs transfected with inhibitor-NC, miR-92a-3p inhibitor, mimics-NC, or miR-92a-3p mimics. Data are reported as means±SD with three replicates. *P<0.05 (ANOVA).

The involved target gene of miR-92a-3p were searched by the bioinformatics software Targetscan (http://www.targetscan.org). As shown in Figure 4A, SIRT6 was a potential target of miR-92a-3p. Subsequently, the results of dual-luciferase reporter assay revealed that overexpression of miR-92a-3p significantly suppressed the luciferase activity of SIRT6 wt reporter, while there was no obvious impact on the luciferase activity of SIRT6 mut group (Figure 4B). We also noticed that inhibition of miR-92a-3p markedly upregulated the expression level of SIRT6 both in mRNA and protein levels, but introducing miR-92a-3p mimics decreased expression of SIRT6 (Figure 4D). Taken together, these data demonstrated that miR-92a-3p negatively regulated SIRT6 expression.

### SIRT6 knockdown restored effects of miR-92a-3p silencing on proliferation and apoptosis in HUVECs exposed to ox-LDL

**Figure 5:**
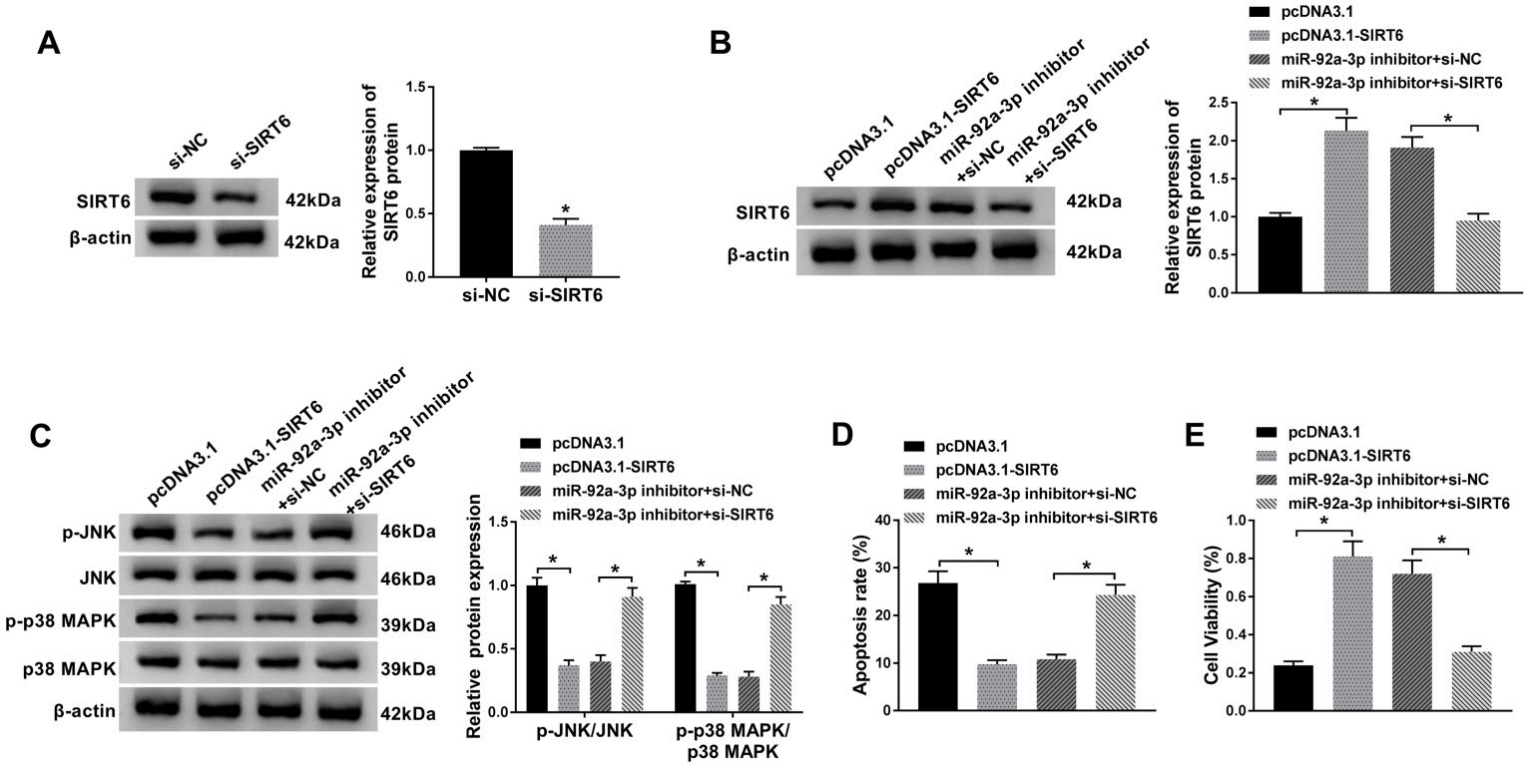
iR-92a-3p was involved in oxidized low-density lipoprotein (ox-LDL)-induced cell apoptosis via targeting SIRT6/MAPK signaling pathway. Human umbilical vein endothelial cells (HUVECs) were treated with 50 µg/mL of ox-LDL for 48 h and transfected with pcDNA3.1, pcDNA3.1-SIRT6, miR-92a-3p inhibitor+anti-negative control (NC), or miR-92a-3p inhibitor+si-SIRT6. **A**-**C**, Western blot assays were carried out to evaluate expression levels of SIRT6, p-JNK/JNK, and p-p38 MAPK/p38 MAPK in transfected HUVECs. **D**, Apoptosis of transfected HUVECs was determined by flow cytometry assay. **E**, MTT analysis was used to assess cell viability of HUVECs post-transfection. Data are reported as means±SD with three replicates. *P<0.05 (ANOVA).

As miR-92a-3p knockdown markedly increased the expression level of SIRT6, we explored the association between miR-92a-3p and SIRT6 in HUVECs exposed to ox-LDL. The knockdown efficiency of SIRT6 was verified by western blot assay (Figure 5A), which suggested that SIRT6 was decreased in si-SIRT6 group compared with si-NC group. Moreover, western blot assay revealed that transfection with pcDNA3.1-SIRT6 or miR-92a-3p inhibitor increased the expression of SIRT6, while transfection with si-SIRT6 counteracted miR-92a-3p-induced effects (Figure 5B). In addition, we found that the ratio of p-JNK/JNK and p-p38 MAPK/p38 MAPK were remarkably decreased in HUVECs with SIRT6 overexpression or miR-92a-3p silencing, while co-transfection with miR-92a-3p inhibitor and si-SIRT6 had opposite results (Figure 5C). The overexpression of SIRT6 inhibited apoptosis of HUVECs; furthermore, SIRT6 knockdown abolished the suppressive effects on apoptosis induced by miR-92a-3p silencing (Figure 5D). As shown in Figure 5E, viability of HUVECs was increased in the SIRT6 overexpression group compared to the control group, but increased cell viability induced by miR-92a-3p silencing was abolished by knockdown of SIRT6. We could conclude that the miR-92a-3p exerted a regulator role in ox-LDL-induced HUVECs at least partially by regulating SIRT6.

## Discussion

In this study, we verified that treatment with 50 µg/mL of ox-LDL for 48 h reduced cell viability and induced apoptosis and oxidative stress of HUVECs, which was attenuated by miR-92a-3p inhibitor or overexpression of SIRT6. In addition, functional experiments further revealed that the knockdown of miR-92a-3p repressed ox-LDL-induced effects on HUVECs by regulating SIRT6/MAPK signaling pathway.

Early studies implied that miRNAs were associated with vascular integrity and cholesterol metabolism (23,24), while the underlying mechanisms involved in the progression of heart disease remain unclear. Therefore, we hypothesized that miR-92a-3p was associated with apoptosis of HUVECs induced by ox-LDL. Circulating members of miR-92a have been suggested to be associated with coronary artery disease (25). Moreover, the miRNA profiling assay conducted by Loyer et al. (26) also confirmed that miR-92a was associated with atherosclerosis; furthermore, it was significantly upregulated and combined with oxidized LDL of low shear stress. Analogously, our results showed that miR-92a-3p was overexpressed in HUVECs treated with ox-LDL. Subsequently, we confirmed that SIRT6 was a targeted gene of miR-92a-3p through bioinformatics and dual-luciferase reporter assay.

Previous studies have shown that ox-LDL induced endothelium cells apoptosis by regulating multiple apoptosis signaling pathways or apoptosis genes, including B-cell CLL/lymphoma 2 (Bcl-2) (27,28). For example, it was reported that ultraviolet B irradiation increased miR-365 expression in NIH3T3 cells (29). Qin et al. (30) reported that miR-365 regulated apoptosis by affecting Bcl-2 expression in HUVECs treated with ox-LDL. Additionally, let-7 mediated apoptosis by regulating oncogenes, including Ras, Myc, HMGA2, and apoptosis-related protein Bcl-xl (31). In agreement with these studies, our results also showed that ox-LDL induced apoptosis of HUVECs in time-dependent and concentration-dependent manners, which was related to the miR-92a-3p/SIRT6 axis. SIRT6 is a member of the nicotinamide adenine dinucleotide-dependent deacetylases. Some studies have shown that SIRT6 plays a vital role in maintaining endothelial function, suggesting that it is a new therapeutic target for atherosclerotic disease (21,32). Additionally, some signaling pathways have already been reported to be associated with apoptosis of endothelial cells induced by ox-LDL, including Notch (33), p38 MAPK (34), and LOX-1-dependent endoplasmic reticulum stress pathway (35). For instance, kaempferol stimulates autophagy to mitigate ox-LDL-induced injury and apoptosis in endothelial cells by inactivating the PI3K/Akt/mTOR pathway (36). The follistatin-related protein protected endothelial cells from ox-LDL-induced apoptosis by affecting the Akt-NF-κB-Bcl-2 pathway *in vitro* and *in vivo* (37). Therefore, we speculated that MAPK pathway might play a role in miR-92a-3p-enhanced apoptosis of ox-LDL-induced endothelial cells.

In summary, we discovered that miR-92a-3p was overexpressed in HUVECs treated with ox-LDL, and functional experiments confirmed that SIRT6 was a target gene of miR-92a-3p. We also proved that the knockdown of miR-92a-3p facilitated the expression of SIRT6 as well as inactivated the MAPK signaling pathway, while miR-92a-3p mimics led to opposite results. Therefore, miR-92a-3p promoted ox-LDL-induced apoptosis of HUVECs by regulating the SIRT6/MAPK signaling pathway.
